# Imaging Pulmonary Embolism in the Era of Subsegmental Clots: What the Internist Needs to Know About Overdiagnosis, Anticoagulation, and Follow-Up

**DOI:** 10.7759/cureus.115885

**Published:** 2026-09-07

**Authors:** Sri Vaibhav R V., Jai C Machur, Sai Tejaswini Goruganthu, Pavan Poojith Nag Chintapalli, Akshaya Priya Govindan

**Affiliations:** 1 Internal Medicine, Karwar Institute of Medical Science, Karwar, IND; 2 Psychiatry, Dr. D. Y. Patil Medical College, Hospital and Research Centre, Pune, IND; 3 Internal Medicine, Kamineni Institute of Medical Sciences, Narketpally, IND; 4 Internal Medicine, Erebuni Medical Academy Foundation, Yerevan, ARM; 5 Internal Medicine, Kanyakumari Government Medical College, Nagercoil, IND

**Keywords:** anticoagulation, multidetector computed tomography, overdiagnosis, pulmonary embolism, subsegmental pulmonary embolism

## Abstract

The widespread adoption of multidetector computed tomography pulmonary angiography has dramatically increased detection of subsegmental pulmonary embolism (SSPE), raising critical questions about overdiagnosis, optimal anticoagulation strategies, and appropriate follow-up. Despite increased detection, observational data demonstrate remarkably low short-term recurrence rates that do not differ significantly from more proximal pulmonary embolism. However, anticoagulation carries major bleeding risks, creating clinical equipoise about net benefit in low-risk isolated SSPE. Radiologist interpretation challenges compound the problem, with a substantial proportion of segmental and subsegmental emboli missed on initial reads in some series. This review presents current evidence on the epidemiology, diagnostic challenges, treatment controversies, and outcome data for SSPE, providing internists with actionable insights for clinical decision-making. Key findings include the lack of randomized controlled trial data to guide anticoagulation decisions, substantial inter-observer variability in SSPE diagnosis, and emerging evidence that selected low-risk patients may be safely observed without anticoagulation.

## Introduction and background

Pulmonary embolism (PE) is a potentially life-threatening condition in which a blood clot (typically originating from deep veins in the legs or pelvis) travels through the venous system and lodges in the pulmonary arterial circulation, obstructing blood flow to the lungs. This obstruction can impair gas exchange and, in severe cases, cause hemodynamic instability or death. PE remains a leading cause of cardiovascular morbidity and mortality, with diagnostic approaches transformed by the introduction of multidetector computed tomography pulmonary angiography (CTPA) in the early 2000s [1-3]. The enhanced spatial resolution of modern CTPA has enabled visualization of increasingly peripheral pulmonary arterial branches, leading to a marked rise in detection of subsegmental pulmonary embolism (SSPE); emboli confined to the fourth-order or more distal pulmonary arterial branches, representing the smallest vessels in the pulmonary arterial tree that are routinely visible on computed tomography imaging (Figure 1) [4-6]. This technological advance has created a clinical paradox: while overall PE diagnosis rates have increased substantially, case-fatality rates have declined, suggesting that many newly detected SSPEs may represent overdiagnosis of clinically insignificant disease [7].

**Figure 1:**
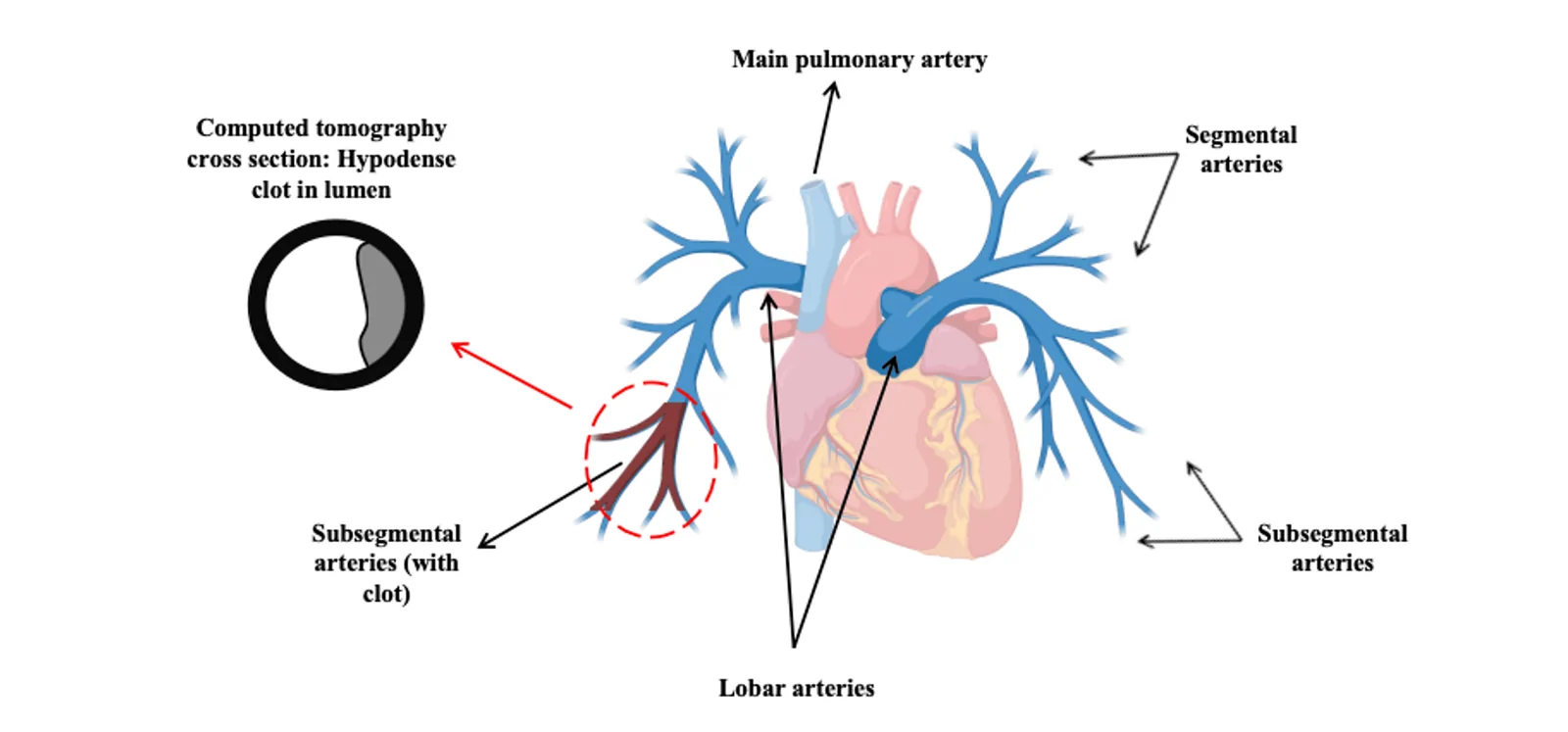
Anatomic schematic and computed tomography appearance of SSPE. The diagram illustrates the pulmonary arterial tree from the main pulmonary artery to subsegmental branches. The axial computed tomography image shows a hypodense thrombus within a subsegmental arterial lumen (red arrow), highlighting the peripheral location and imaging characteristics of isolated subsegmental pulmonary embolism (SSPE). The image was created by the authors using BioRender (BioRender, Toronto, Ontario, Canada).

The rationale for anticoagulation in PE is to prevent thrombus extension, reduce the risk of recurrent venous thromboembolism (VTE), and lower mortality. For patients with proximal PE (involving the main, lobar, or segmental pulmonary arteries), randomized trials have unequivocally demonstrated that anticoagulation reduces recurrent VTE and death. However, this benefit comes at a cost: anticoagulant therapy increases the risk of major bleeding, including intracranial hemorrhage and gastrointestinal bleeding, with rates that can reach up to 5% in some populations. The central dilemma with SSPE is that the natural history of these small, peripheral clots is poorly understood; they may resolve spontaneously without intervention, yet there are no large randomized trials to confirm that withholding anticoagulation is safe (Figure 2) [8]. Contemporary observational data suggest that isolated SSPE accounts for approximately 10% of all diagnosed PE cases [9], yet management approaches vary widely across institutions and providers, reflecting fundamental uncertainty about optimal care.

**Figure 2:**
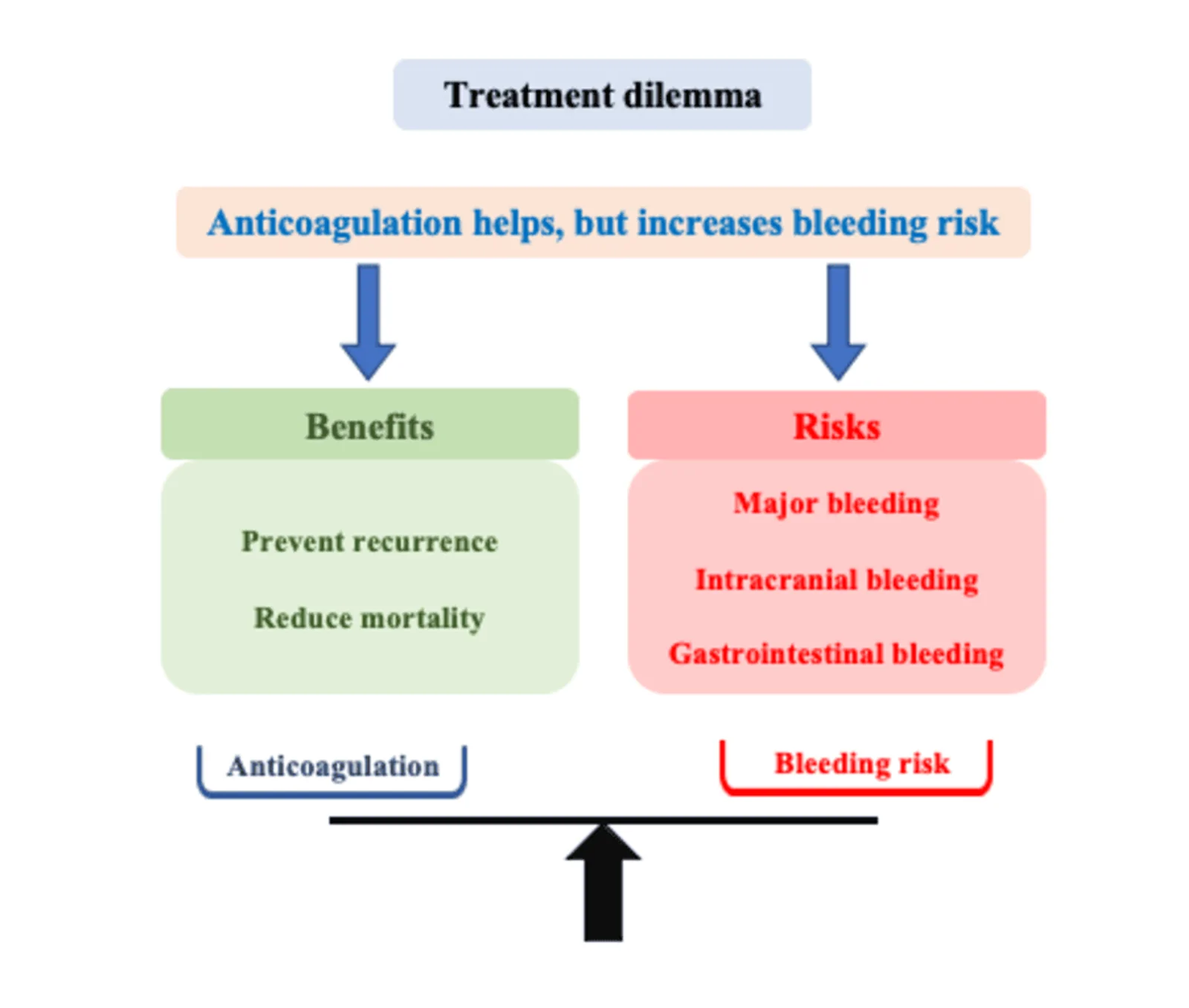
Treatment dilemma in SSPE. SSPE: subsegmental pulmonary embolism The image was created by the authors using Microsoft PowerPoint (Microsoft Corporation, Redmond, Washington, USA).

The clinical significance of isolated SSPE (defined as subsegmental emboli without concurrent proximal thrombus or documented deep vein thrombosis (DVT)) remains uncertain [10]. This review presents current evidence on three critical domains for internists managing patients with suspected or confirmed SSPE: (i) the scope and mechanisms of overdiagnosis, including imaging technology limitations and diagnostic accuracy challenges; (ii) anticoagulation decision-making, including recurrence and bleeding risks, trial evidence, and risk stratification approaches; and (iii) follow-up strategies, including surveillance protocols, long-term complication rates, and outcomes in special populations. By integrating data from observational cohorts, systematic reviews, and ongoing trial protocols, this report aims to provide evidence-based guidance for clinical practice while highlighting critical knowledge gaps that require further research.

## Review

Literature search methodology

This narrative review was conducted by systematically searching PubMed, Scopus, and Google Scholar databases from January 2014 to March 2026 using the following search terms and Boolean operators: (‘subsegmental pulmonary embolism’ OR ‘SSPE’ OR ‘isolated subsegmental PE’) AND (‘anticoagulation’ OR ‘overdiagnosis’ OR ‘CTPA’ OR ‘recurrence’ OR ‘bleeding’ OR ‘follow-up’ OR ‘surveillance’). Additional articles were identified through hand-searching reference lists of included studies, systematic reviews, and relevant guidelines. We included observational cohorts, systematic reviews, meta-analyses, randomized trial protocols, and major society guidelines that addressed SSPE diagnosis, management, or outcomes. Studies were excluded if they did not specifically address subsegmental PE or if they focused exclusively on proximal PE without differentiation. The evidence synthesis was narratively organized to highlight key themes, evidence gaps, and clinical implications for internists.

The epidemiology of SSPE

Prevalence and Detection Rates

The reported prevalence of SSPE varies substantially across clinical settings, imaging indications, and patient populations. In a large cardiac CT cohort screened between 2021 and 2023, incidental PE was detected in 1% of all coronary CT angiography scans; among confirmed PE cases, 45.5% of emboli that were missed on initial radiology reports involved segmental or subsegmental arteries [11]. This finding underscored both the frequency of peripheral emboli and the diagnostic challenges they pose.

In COVID-19 cohorts, subsegmental location was recorded in 17% of ancestral-strain PE cases, with proportions ranging from 0% to 19% across different viral variants [12]. A cancer center CTPA cohort of 8,023 patients found that 14% had PE; among the small subgroup with normal D-dimer and imaging-proven PE, 66% had segmental or subsegmental PE (not exclusively isolated SSPE) [13]. These data illustrated the heterogeneity of SSPE prevalence across clinical contexts and the particular relevance of subsegmental emboli in cancer populations and during acute viral illness.

Systematic reviews have attempted to synthesize SSPE prevalence data but consistently report substantial heterogeneity across included studies, preventing reliable pooled estimates [14]. The proportion of all diagnosed PE that is isolated SSPE (without concurrent proximal thrombus) is estimated at approximately 10% in contemporary series [10], though this figure varies with imaging protocols, patient selection, and radiologist interpretation practices.

Temporal Trends and the CTPA Era

The introduction and widespread adoption of multidetector CTPA has been repeatedly identified as the primary driver of increased SSPE detection [15-17]. Health technology assessment reports and trial planning documents note a clear temporal association between CTPA dissemination and rising SSPE diagnosis rates, accompanied by falling PE case-fatality rates [7]. This epidemiologic pattern increased diagnosis without a proportional increase in mortality is a hallmark of potential overdiagnosis and has motivated calls for randomized trials to determine whether anticoagulation is necessary for all detected SSPEs.

The shift from ventilation-perfusion scanning to CTPA as the primary PE diagnostic modality has fundamentally altered the spectrum of diagnosed disease [15]. Earlier imaging technologies lacked the spatial resolution to reliably detect subsegmental emboli, meaning that many cases now diagnosed as SSPE would have been missed in the pre-CTPA era [18]. Whether these previously undetected emboli caused clinically significant harm remains a central question in contemporary PE management.

Overdiagnosis: imaging technology and diagnostic challenges

Mechanisms of Overdiagnosis

Overdiagnosis in SSPE occurs through multiple mechanisms: (i) detection of emboli that would never cause symptoms or adverse outcomes (true overdiagnosis); (ii) false-positive interpretations of imaging artifacts or normal anatomic variants as emboli; and (iii) detection of emboli that would spontaneously resolve without intervention [19-21]. The relative contribution of each mechanism remains uncertain, but available evidence suggests all three play important roles.

The enhanced sensitivity of modern CTPA comes at the cost of reduced specificity for clinically significant disease [22]. Multidetector scanners with 64 or more detector rows can visualize fourth-order and more distal pulmonary arterial branches with high spatial resolution, but image quality in these peripheral vessels is often degraded by motion artifacts, beam hardening, and partial volume effects [23,24]. These technical limitations create opportunities for false-positive interpretations, particularly when radiologists are primed to search for PE in symptomatic patients.

Diagnostic Accuracy and Inter-observer Variability

Diagnostic accuracy for SSPE is substantially lower than for proximal PE, with marked inter-observer variability documented across multiple studies [25]. In the previously described cardiac CT cohort, 45.5% of segmental and subsegmental emboli confirmed by blinded expert review were missed in the initial clinical radiology report [11]. This high miss rate reflects both the technical difficulty of interpreting peripheral pulmonary arteries and the lower clinical suspicion for PE in patients undergoing cardiac CT for other indications.

Single-center chart reviews and retrospective series consistently describe substantial disagreement between radiologists in SSPE diagnosis, with re-reads frequently changing classification [7,14]. This limited reproducibility raises concerns about both false-positive and false-negative diagnoses at the subsegmental level. However, the available literature does not provide robust, generalizable numeric estimates of false-positive rates for SSPE on CTPA, representing a critical evidence gap.

Incidental Detection

Incidental PE emboli detected on imaging performed for other clinical indications represent a growing proportion of SSPE diagnoses [26,27]. In the cardiac CT screening cohort, incidental PE was found in approximately 1% of scans, with nearly half of initially missed cases involving segmental or subsegmental vessels [11]. Cancer imaging presents a particularly high-yield context for incidental SSPE detection; in one cancer center cohort, 14% of patients undergoing CTPA had PE, and among the uncommon subset with normal D-dimer and imaging-proven PE, 66% of cases were segmental or subsegmental [13].

The clinical significance of incidental SSPE is especially uncertain. These patients, by definition, lack symptoms directly attributable to PE, raising questions about whether detected emboli represent clinically meaningful disease or incidental findings of uncertain significance. Multiple trial protocols now focus specifically on isolated SSPE as a distinct management dilemma, recognizing that incidental and isolated subsegmental emboli may warrant different treatment approaches than symptomatic proximal PE [7,28].

Artificial Intelligence (AI) and Automated Detection

AI and automated detection tools are being developed to reduce interpretation variability and improve SSPE detection rates [29-31]. In the cardiac CT cohort, AI-assisted review identified subsegmental emboli that had been missed on initial clinical reads [11]. While these tools may improve sensitivity and reduce inter-observer variability, their clinical impact remains under evaluation. Increased sensitivity without improved specificity could paradoxically worsen overdiagnosis by detecting more emboli of uncertain clinical significance. Table 1 summarizes key evidence on overdiagnosis of SSPE, including detection rates, miss rates, and variability across clinical contexts such as cardiac CT, COVID-19, and cancer populations.

**Table 1:** Evidence on overdiagnosis of SSPE. PE: pulmonary embolism; CCTA: coronary computed tomography angiography; CTPA: computed tomography pulmonary angiography; SSPE: subsegmental pulmonary embolism

Study population and design	Key finding	Numeric estimate	Clinical implication	Population type	Ref.
Cardiac CT cohort (2021 to 2023), retrospective review with AI-assisted re-read	Incidental PE detection rate and miss rate for subsegmental emboli	Incidental PE in 1% of CCTA scans; 45.5% of segmental/subsegmental emboli missed on initial report	High miss rate suggests both underdiagnosis and potential for false positives; AI may improve detection but clinical significance uncertain	Segmental/subsegmental (mixed)	[11]
COVID-19 CTPA cohort (March 2020 to Feb 2022), observational series	Proportion of PE that is subsegmental across viral variants	17% of ancestral-strain PE cases were subsegmental (range 0 to 19% across variants)	Substantial proportion of COVID-associated PE involves peripheral vessels; clinical significance may differ from non-COVID PE	Segmental/subsegmental (mixed)	[12]
Cancer center CTPA cohort (8,023 patients), retrospective analysis	PE prevalence and distribution in cancer patients with normal D-dimer	14% had PE; among normal-D-dimer PE cases, 66% were segmental/subsegmental	Cancer patients have high rates of peripheral PE even with normal D-dimer; challenges traditional diagnostic algorithms	Segmental/subsegmental (mixed)	[13]
Systematic review of SSPE outcomes, multiple observational cohorts	Heterogeneity in reported SSPE prevalence and outcomes	Wide variation across studies; no reliable pooled prevalence estimate	Substantial heterogeneity limits generalizability; population-specific estimates needed	Isolated SSPE and mixed populations	[14]
Health technology assessment and trial planning documents	Temporal trends in SSPE diagnosis and PE case-fatality	Clear increase in SSPE diagnoses since widespread CTPA adoption; falling PE case-fatality despite rising incidence	Epidemiologic pattern consistent with overdiagnosis; motivates trials of withholding anticoagulation	Isolated SSPE and mixed populations	[7]
Contemporary PE epidemiology analysis	Proportion of all PE that is isolated SSPE	Approximately 10% of diagnosed PE cases are isolated SSPE	Isolated SSPE represents a substantial and growing proportion of PE diagnoses requiring management decisions	Isolated SSPE	[10]

Anticoagulation: evidence, risks, and controversies

Current Practice Patterns

Despite limited high-quality evidence supporting anticoagulation for isolated SSPE, most patients with SSPE receive anticoagulation in contemporary practice [32,33]. Multicenter institutional reviews document widespread anticoagulation use, reflecting clinician concern about recurrent VTE and extrapolation from proximal PE management guidelines [34]. However, observational syntheses and guideline reviews describe substantial practice variation, with management strategies ranging from full-dose anticoagulation to clinical observation without treatment [14]. This heterogeneity in practice reflects fundamental uncertainty about the balance of benefits and harms. The absence of randomized controlled trial data has left management decisions to individual clinician judgment, institutional protocols, and patient preferences.

Recurrent VTE Risk With Anticoagulation

Available observational data suggest low short-term recurrent VTE rates in anticoagulated SSPE patients [35,36]. Trial planning documents cite a commonly referenced 90-day recurrent VTE risk of approximately 0.8% in pooled observational summaries of anticoagulated, low-risk isolated SSPE patients [28]. In one multicenter observational cohort, 90-day recurrent VTE was 0.9% for isolated SSPE versus 1.8% for proximal PE (p = 0.458) [37]. At one year, the same cohort reported recurrent VTE rates of 2.7% for isolated SSPE versus 6.3% for proximal PE (p = 0.126), with a subdistribution hazard ratio of 0.84 (95% CI 0.19 to 3.60) [37]. These low absolute recurrence rates raise questions about whether anticoagulation provides meaningful benefit, particularly when balanced against bleeding risks.

Major Bleeding Risk

Anticoagulation carries nontrivial bleeding risk that must be weighed against potential VTE prevention benefits [38-40]. Major bleeding rates in anticoagulated patients reach up to approximately 5% in some series [28]. This risk is not specific to SSPE but reflects the general hazards of therapeutic anticoagulation in populations that include elderly patients, those with comorbidities, and individuals at elevated bleeding risk.

The critical clinical question is whether recurrence rates in anticoagulated SSPE patients (approximately 0.8% at 90 days) would be substantially higher without anticoagulation, and whether any increase would exceed the major bleeding risk. Systematic reviews report low recurrence rates in selected non-anticoagulated patients but stress that estimates are limited by observational designs and selection bias [14]. Precise comparative recurrence rates for treated versus untreated SSPE remain imprecise and subject to substantial heterogeneity across studies.

Anticoagulant Type and Duration

There is insufficient SSPE-specific randomized evidence to support differential efficacy or safety of direct oral anticoagulants versus low-molecular-weight heparin or warfarin. Systematic reviews highlight the absence of SSPE-specific comparative data for different anticoagulant classes [14,41]. In practice, anticoagulant selection for SSPE typically follows general PE guidelines, with direct oral anticoagulants increasingly preferred based on convenience, safety, and efficacy data from proximal PE trials. A three-month anticoagulation course is commonly used as the standard comparator in trial protocols [7,28]. This duration reflects guideline recommendations for provoked PE, though optimal anticoagulation duration for SSPE remains unknown beyond the pragmatic three-month standard.

Risk Stratification Tools

General PE risk stratification tools such as the Pulmonary Embolism Severity Index and Wells score are applied in clinical practice and reviewed in PE risk-assessment literature [42]. However, these tools have not been well validated specifically for isolated SSPE [14], representing an important gap in clinical decision support. Most SSPE patients are hemodynamically stable and have low Pulmonary Embolism Severity Index scores, making it difficult to discriminate risk within this already low-risk population [43,44]. Novel risk stratification approaches that incorporate imaging characteristics (e.g., clot burden, right ventricular strain), biomarkers, and clinical factors may be needed to identify the subset of SSPE patients who truly benefit from anticoagulation [45,46].

Provoked Versus Unprovoked SSPE

Data are limited on differential treatment rates and outcomes for provoked (e.g., cancer-associated, postoperative) versus unprovoked SSPE. Observational series note that many incidental SSPEs are detected in cancer patients, indicating frequent SSPE occurrence in this high-risk population [13]. However, robust pooled estimates comparing anticoagulation rates or outcomes for provoked versus unprovoked SSPE are lacking [14].

Cancer-associated SSPE presents particular management challenges, as these patients face both elevated VTE recurrence risk and increased bleeding risk from anticoagulation [47-49]. The balance of benefits and harms may differ substantially from unprovoked SSPE in otherwise healthy individuals, yet evidence to guide differential management is scarce. Similarly, data on SSPE in other special populations (pregnancy [50], recent surgery [51], prolonged immobilization) are insufficient to support population-specific recommendations.

Net Clinical Benefit

Formal net clinical benefit modeling specifically focused on SSPE (balancing recurrent VTE avoided versus anticoagulant bleeding harms) has not been performed due to the absence of untreated recurrence rate data. Systematic reviews call for randomized data to permit such analyses [41]. The approximately 0.8% 90-day recurrent VTE risk with anticoagulation and up to 5% major bleeding risk suggest a narrow therapeutic margin, but without data on untreated recurrence rates, definitive benefit-harm quantification is not possible. Table 2 synthesizes available evidence on anticoagulation for SSPE, highlighting recurrence and bleeding rates, observational comparisons with proximal PE, and current gaps in randomized trial data.

**Table 2:** Evidence on anticoagulation for SSPE. VTE: venous thromboembolism; SSPE: subsegmental pulmonary embolism; PE: pulmonary embolism; CI: confidence interval; ESC: European Society of Cardiology; CTPA: computed tomography pulmonary angiography

Study design and population	Outcome measured	Numeric estimate	Comparator/context	Clinical implication	Population type	Ref.
SAFE-SSPE trial protocol, literature review for trial design	90-day recurrent VTE in anticoagulated low-risk isolated SSPE	Approximately 0.8%	Background rate used for trial power calculation	Low recurrence rate with anticoagulation; unclear if anticoagulation is necessary	Isolated SSPE	[28]
SAFE-SSPE trial protocol, literature review for trial design	Major bleeding in anticoagulated patients	Up to approximately 5%	Background rate used for trial power calculation	Nontrivial bleeding risk must be weighed against VTE prevention benefit	Isolated SSPE	[28]
Multicenter observational cohort (334 PE patients, 111 isolated SSPE)	90-day recurrent VTE	SSPE 0.9% vs proximal PE 1.8% (p = 0.458)	Direct comparison of isolated SSPE to more proximal PE	No significant difference in short-term recurrence; questions benefit of anticoagulation for SSPE	Isolated SSPE	[37]
Same multicenter observational cohort	One-year recurrent VTE	SSPE 2.7% vs proximal PE 6.3% (p = 0.126)	Direct comparison with subdistribution hazard ratio 0.84 (95% CI 0.19 to 3.60)	Trend toward lower recurrence in SSPE but not statistically significant; low absolute rates	Isolated SSPE	[37]
Systematic review of SSPE outcomes, multiple observational cohorts	Recurrent VTE in non-anticoagulated selected patients	Low absolute rates but imprecise estimates	Observational data with selection bias	Suggests some patients may be safely observed, but evidence quality is low	Isolated SSPE and mixed populations	[14]
Cochrane systematic review update	Randomized controlled trial evidence for SSPE anticoagulation	-	Comprehensive systematic review	Definitive evidence lacking; current practice based on extrapolation and observational data	Isolated SSPE	[41]
Multicenter institutional review	Anticoagulation use patterns	Most SSPE patients receive anticoagulation in contemporary practice	Observational practice patterns	Widespread anticoagulation despite limited evidence reflects clinical uncertainty	Isolated SSPE	[34]
STOPAPE trial protocol	Standard anticoagulation duration	Three-month course used as comparator	Trial design for withholding vs giving anticoagulation	Three months is pragmatic standard but optimal duration for SSPE unknown	Isolated SSPE	[7]
ESC PE guidelines and risk stratification review	Risk stratification tools (PE Severity Index, Wells)	Applied in general PE populations	General PE risk assessment	Tools not validated specifically for isolated SSPE; most SSPE patients are low-risk by standard criteria	Isolated SSPE and mixed populations	[42]
Cancer center CTPA cohort	SSPE in cancer patients	High proportion of incidental PE in cancer imaging; 66% of normal-D-dimer PE were segmental/subsegmental	Cancer population with elevated baseline VTE risk	Cancer-associated SSPE may warrant different management, but evidence is limited	Segmental/subsegmental (mixed)	[13]

Follow-up strategies and long-term outcomes

Recurrence Rates and Comparative Outcomes

As detailed in the preceding section, short-term recurrence after isolated SSPE appears low and not clearly different from more proximal PE in available observational cohorts [52]. The same multicenter study cited earlier reported 90-day recurrent VTE of 0.9% for isolated SSPE versus 1.8% for proximal PE and one-year recurrent VTE of 2.7% versus 6.3%, respectively [37]. These data suggest that isolated SSPE may have a more benign natural history than proximal PE, though the observational design and potential for selection bias limit causal inference.

Six-month recurrence data are not consistently reported in the available literature, representing a gap in understanding the time course of recurrence risk. The available evidence suggests that most recurrences occur within the first three months, consistent with general VTE recurrence patterns, but SSPE-specific temporal patterns have not been well characterized.

Mortality Comparisons

Reported comparative mortality data are limited but suggest no significant difference between isolated SSPE and more proximal PE after adjustment for confounders. In the multicenter observational cohort of 334 PE patients (111 with isolated SSPE), one-year mortality did not differ significantly between isolated SSPE and proximal PE after adjustment for frailty and other variables (adjusted hazard ratio 1.72, 95% confidence interval (CI) 0.92 to 3.23) [37]. The wide CI reflects limited statistical power but suggests that any mortality difference is likely small. Thirty-day and 90-day mortality comparisons are not reliably reported in the available literature. The overall low mortality in SSPE cohorts makes it difficult to detect differences and raises questions about whether anticoagulation meaningfully reduces mortality in this population. The falling PE case-fatality rates observed in parallel with rising SSPE detection further suggest that many detected SSPEs may not be life-threatening [7].

Progression and Imaging Surveillance

The available literature does not provide robust, generalizable rates for progression from SSPE to proximal PE or development of new DVT during follow-up. Existing studies report overall recurrent VTE event counts but do not uniformly break out progression patterns [37]. This represents an important knowledge gap, as progression to more extensive thrombosis would support more aggressive anticoagulation, while spontaneous resolution would support observation.

Consensus imaging surveillance protocols specific to SSPE are not established. Available studies and trial protocols highlight clinical equipoise and describe designs that follow patients clinically with symptom-driven imaging rather than routine scheduled repeat CTPA or serial leg ultrasonography [9]. The lack of standardized surveillance algorithms reflects both the uncertainty about SSPE natural history and concerns about radiation exposure and cost from routine repeat imaging.

Multicenter prospective data on non-anticoagulated SSPE management exist but do not define precise imaging schedules in the available summaries [9]. Clinical practice varies widely, with some centers performing routine repeat imaging at one to three months and others relying entirely on clinical follow-up. The optimal surveillance strategy likely depends on individual patient risk factors, symptom evolution, and shared decision-making.

Long-Term Complications

Data on post-PE syndrome, chronic thromboembolic pulmonary hypertension (CTEPH), and quality of life outcomes specifically for SSPE are limited. A general review of CTEPH in PE survivors reports an overall incidence around 3% among PE survivors [53], but SSPE-specific rates are not reported in the available literature. Given the peripheral location and typically small clot burden of SSPE, the risk of CTEPH may be lower than for proximal PE, but this hypothesis has not been rigorously tested.

Post-PE syndrome, characterized by persistent dyspnea, exercise intolerance, and reduced quality of life after PE, has not been systematically studied in SSPE populations [54-56]. Validated long-term quality of life measures specifically for SSPE patients are not available in the literature, representing a significant gap in understanding the full clinical impact of SSPE diagnosis and treatment. Whether the diagnosis itself, anticoagulation treatment, or the underlying embolic event drives long-term symptoms remains unclear. Table 3 compiles follow-up and long-term outcome data for SSPE, including short- and long-term recurrence, mortality, surveillance approaches, and unresolved questions about chronic complications.

**Table 3:** Evidence on follow-up and long-term outcomes after SSPE. PE: pulmonary embolism; SSPE: subsegmental pulmonary embolism; VTE: venous thromboembolism; HR: hazard ratio; CI: confidence interval; CTPA: computed tomography pulmonary angiography; CTEPH: chronic thromboembolic pulmonary hypertension; DVT: deep vein thrombosis; QoL: quality of life

Study design and population	Outcome measured	Numeric estimate	Comparator/context	Clinical implication	Population type	Ref.
Multicenter observational cohort (334 PE patients, 111 isolated SSPE)	90-day recurrent VTE	SSPE 0.9% vs proximal PE 1.8% (p = 0.458)	Direct comparison of isolated SSPE to proximal PE	Low short-term recurrence; no significant difference from proximal PE	Isolated SSPE	[37]
Same multicenter observational cohort	One-year recurrent VTE	SSPE 2.7% vs proximal PE 6.3% (p = 0.126)	Subdistribution hazard ratio 0.84 (95% CI 0.19 to 3.60)	Low one-year recurrence; trend toward lower risk than proximal PE but not significant	Isolated SSPE	[37]
Same multicenter observational cohort	One-year mortality	Adjusted HR 1.72 (95% CI 0.92 to 3.23)	Comparison of isolated SSPE to proximal PE after adjustment for frailty and covariates	No significant mortality difference after adjustment; wide CI reflects limited power	Isolated SSPE	[37]
Prospective multicenter studies of non-anticoagulated SSPE	Clinical follow-up approach	Symptom-driven imaging rather than routine scheduled repeat CTPA	Observational management without anticoagulation	Supports clinical surveillance approach but optimal imaging schedule undefined	Isolated SSPE	[9]
General CTEPH review in PE survivors	CTEPH incidence	Approximately 3% overall in PE survivors	General PE population, not SSPE-specific	SSPE-specific CTEPH rates unknown; may be lower given peripheral location and small clot burden	General PE (not SSPE-specific)	[53]
Systematic review of SSPE outcomes	Progression to proximal PE or new DVT	Specific progression rates not reported; overall recurrent VTE events documented	Heterogeneous observational cohorts	Progression patterns poorly characterized; important gap for understanding natural history	Isolated SSPE and mixed populations	[14]
Contemporary PE epidemiology analysis	Proportion of PE that is isolated SSPE	Approximately 10% of diagnosed PE cases	Contemporary CTPA era	Substantial and growing proportion of PE diagnoses requiring follow-up decisions	Isolated SSPE	[10]
Observational reviews of SSPE management	Quality of life and post-PE syndrome	No validated long-term QoL measures reported for SSPE	Evidence gap	Impact of SSPE diagnosis and treatment on long-term QoL unknown	Isolated SSPE	[14]

Special populations and clinical contexts

Cancer-Associated SSPE

Cancer patients represent a particularly important population for SSPE management, as they have both elevated VTE risk and increased bleeding risk from anticoagulation [10]. In the cancer center CTPA cohort, 14% of patients had PE, and among the small subgroup with normal D-dimer and imaging-proven PE, 66% had segmental or subsegmental PE [13]. This high proportion of peripheral emboli in cancer patients challenges traditional diagnostic algorithms that rely on D-dimer testing.

Recent studies have specifically examined lung cancer-associated SSPE [57], but the available abstracts do not provide reproducible numeric outcome rates for SSPE in cancer populations. The balance of anticoagulation benefits and harms may differ substantially in cancer patients compared to the general population, given their elevated baseline VTE recurrence risk (approximately 15% to 20% annually in some cancer types) and bleeding risk from thrombocytopenia, tumor invasion of vessels, and concurrent antiplatelet therapy.

Current guidelines generally recommend anticoagulation for cancer-associated VTE regardless of clot location, but whether this approach is optimal for isolated SSPE in cancer patients remains uncertain. The duration of anticoagulation in cancer-associated SSPE is also unclear, with some experts recommending indefinite anticoagulation while cancer is active and others suggesting time-limited courses based on cancer type and treatment status.

COVID-19-Associated SSPE

The COVID-19 pandemic highlighted the importance of SSPE in acute viral illness. In one COVID-19 CTPA cohort, subsegmental location was recorded in 17% of ancestral-strain PE cases, with proportions varying across different viral variants [12]. The pathophysiology of COVID-19-associated PE may differ from traditional VTE, with in situ pulmonary microthrombosis potentially playing a larger role than embolization from lower-extremity DVT.

The clinical significance of COVID-19-associated SSPE and optimal management remain uncertain. Some experts have questioned whether these peripheral emboli represent true PE requiring anticoagulation or rather localized pulmonary microthrombosis that might resolve spontaneously. Observational data on outcomes are limited, and management approaches have varied widely across institutions and over the course of the pandemic.

Incidental SSPE in Cardiac Imaging

The increasing use of cardiac CT for coronary artery disease evaluation has led to incidental detection of PE in approximately 1% of scans, with nearly half of initially missed cases involving segmental or subsegmental vessels [11]. These patients typically lack symptoms attributable to PE and are undergoing imaging for entirely different indications, raising questions about the clinical significance of detected emboli.

AI tools are being developed to improve detection of incidental PE on cardiac CT [11], but whether increased detection improves patient outcomes remains unknown. The risk of overdiagnosis and overtreatment may be particularly high in this population, as many detected emboli might never cause symptoms or adverse outcomes if left undetected.

Practical recommendations for internists

A four-step clinical framework for internists encompassing risk assessment, risk-stratified management, shared decision-making, and close clinical follow-up with reassessment if symptoms change (Figure 3).

**Figure 3:**
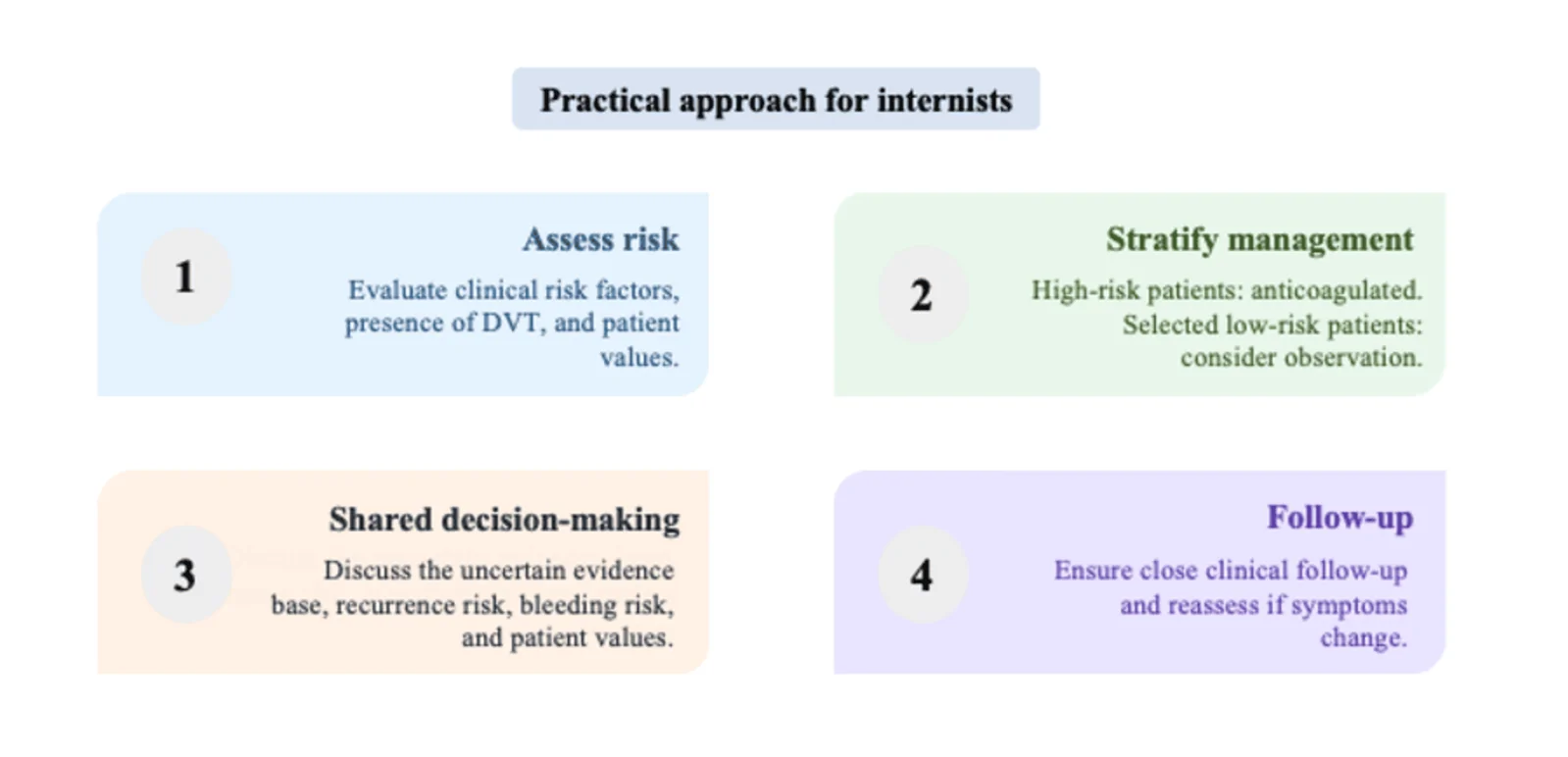
Practical approach to the management of SSPE. SSPE: subsegmental pulmonary embolism; DVT: deep vein thrombosis The image was created by the authors using Microsoft PowerPoint (Microsoft Corporation, Redmond, Washington, USA).

Diagnostic Approach

When SSPE is suspected or incidentally detected, internists should: (i) Confirm the diagnosis: Given the high inter-observer variability in SSPE interpretation, consider expert radiology review or second-read for isolated subsegmental findings, particularly when clinical suspicion is low or imaging quality is suboptimal. (ii) Assess clinical context: Distinguish between symptomatic SSPE (patient presenting with symptoms potentially attributable to PE) and incidental SSPE (emboli detected on imaging performed for other indications). Clinical context should inform management decisions. (iii) Evaluate for proximal thrombus and DVT: Ensure that reported SSPE is truly isolated (no concurrent segmental or more proximal PE) and consider lower-extremity ultrasound to exclude DVT, as the presence of proximal thrombus or DVT would support anticoagulation. (iv) Consider alternative diagnoses: In patients with atypical presentations or low clinical probability, consider whether imaging findings might represent artifacts or normal variants rather than true emboli.

Risk Stratification

For confirmed isolated SSPE, assess individual patient risk factors: (i) Bleeding risk: Evaluate for factors that increase bleeding risk with anticoagulation (age >75 years, prior bleeding, thrombocytopenia, renal or hepatic impairment, concurrent antiplatelet therapy, recent surgery or trauma, fall risk). (ii) VTE recurrence risk: Consider factors that may increase recurrence risk (active cancer, prior VTE, unprovoked presentation, inherited thrombophilia, persistent risk factors). (iii) Cardiopulmonary reserve: Assess baseline cardiopulmonary function, as patients with limited reserve may be more vulnerable to adverse outcomes from even small emboli. (iv) Clinical stability: Hemodynamic stability, oxygen saturation, and symptom severity should inform urgency and intensity of treatment.

Treatment Decisions

Given the current evidence base, reasonable approaches include: (i) Anticoagulation for higher-risk patients: Consider full-dose anticoagulation for patients with active cancer, prior VTE, unprovoked SSPE, persistent risk factors, limited cardiopulmonary reserve, or high VTE recurrence risk by clinical assessment. (ii) Observation for lower-risk patients: In carefully selected low-risk patients (hemodynamically stable, good cardiopulmonary reserve, provoked SSPE with resolved risk factor, high bleeding risk, strong patient preference to avoid anticoagulation), clinical observation without anticoagulation may be reasonable with close follow-up. (iii) Shared decision-making: Given the uncertainty in the evidence base, engage patients in shared decision-making that explicitly discusses the low recurrence risk (approximately 0.9% at 90 days with anticoagulation), uncertain benefit of anticoagulation, bleeding risk (up to 5%), and individual patient values and preferences. (iv) Anticoagulant selection: If anticoagulation is chosen, direct oral anticoagulants are generally preferred over warfarin based on convenience, safety, and efficacy data from general PE populations, unless contraindicated (e.g., severe renal impairment, mechanical heart valves, antiphospholipid syndrome). (v) Duration: A three-month course is a reasonable standard for provoked SSPE with resolved risk factors. For unprovoked SSPE or persistent risk factors, consider extended anticoagulation with reassessment at three to six months based on bleeding risk and patient preferences.

Follow-Up Strategy

For patients with isolated SSPE, whether anticoagulated or observed: (i) Clinical follow-up: Schedule follow-up within one to two weeks to assess symptom evolution, treatment tolerance, and adherence. Educate patients about symptoms of recurrent VTE (new or worsening dyspnea, chest pain, leg swelling) and bleeding complications. (ii) Imaging surveillance: Routine repeat CTPA is not recommended for asymptomatic patients. Consider repeat imaging only if symptoms worsen or new symptoms develop. For patients managed without anticoagulation, lower the threshold for repeat imaging if symptoms evolve. (iii) Lower-extremity ultrasound: Consider baseline and/or follow-up lower-extremity ultrasound, particularly in patients managed without anticoagulation, to exclude DVT that would support anticoagulation. (iv) Reassessment: At three months, reassess need for continued anticoagulation based on risk factor resolution, bleeding events, patient preferences, and evolving evidence. (v) Long-term monitoring: Educate patients about long-term VTE recurrence risk and symptoms. For patients with unprovoked SSPE who discontinue anticoagulation, consider periodic reassessment of recurrence risk and discussion of resuming anticoagulation if risk factors evolve.

Documentation and communication

Clear documentation and communication are essential: (i) Document decision-making: Record the rationale for anticoagulation or observation decisions, including risk-benefit assessment and shared decision-making discussion. (ii) Communicate with radiologists: If SSPE diagnosis is uncertain, communicate with interpreting radiologists to clarify findings and consider expert review. (iii) Coordinate care: For patients with cancer, recent surgery, or other complex medical issues, coordinate management with relevant specialists (oncology, surgery, hematology). (iv) Patient education: Provide clear written and verbal instructions about treatment plan, follow-up schedule, and warning symptoms requiring urgent evaluation.

Future directions and ongoing trials

Several randomized trials are addressing the critical question of whether anticoagulation is necessary for isolated SSPE: (i) SAFE-SSPE trial: This multicenter randomized non-inferiority trial compares clinical surveillance (no anticoagulation) versus anticoagulation in low-risk isolated SSPE. The protocol cites a 90-day recurrent VTE baseline of approximately 0.8% and major bleeding risks up to 5% to power the study [28]. Results from this trial will provide the first high-quality randomized evidence to guide management decisions. (ii) STOPAPE trial: This randomized trial protocol examines withholding anticoagulation versus full anticoagulation for isolated or incidental SSPE [7]. A methodological commentary documents the challenges encountered in trial design and recruitment [7], highlighting the practical difficulties of conducting rigorous research in this area.

Key research priorities to address current evidence gaps include: (i) Diagnostic accuracy studies: Rigorous studies of inter-observer agreement, false-positive rates, and clinical validation of SSPE diagnosis are needed. Comparison of expert radiologist interpretation to AI-assisted detection may help define optimal diagnostic approaches. (ii) Natural history studies: Prospective cohorts of untreated or minimally treated SSPE patients with systematic follow-up imaging and clinical assessment would clarify progression rates, spontaneous resolution, and true recurrence risk. (iii) Risk stratification tools: Development and validation of SSPE-specific risk stratification tools that incorporate imaging characteristics, biomarkers, and clinical factors could identify patients who truly benefit from anticoagulation. (iv) Long-term outcomes: Studies of post-PE syndrome, CTEPH, quality of life, and functional outcomes specifically in SSPE populations would clarify the full clinical impact of SSPE diagnosis and treatment. (v) Special populations: Dedicated studies in cancer patients, pregnant women, postoperative patients, and other special populations are needed to guide population-specific management. (vi) Net clinical benefit modeling: Once randomized trial data are available, formal decision analyses and net clinical benefit models can quantify the balance of anticoagulation benefits and harms across different patient subgroups. (vii) Implementation science: Research on optimal strategies for shared decision-making, patient education, and implementation of evidence-based SSPE management in diverse clinical settings will be important for translating trial results into practice.

Advances in imaging technology will continue to shape SSPE diagnosis and management: (i) AI: AI-assisted detection tools may improve sensitivity and reduce inter-observer variability, but their impact on clinical outcomes requires evaluation. Increased detection without improved specificity could worsen overdiagnosis; (ii) Dual-energy CT: Dual-energy CTPA with iodine mapping may improve diagnostic accuracy for peripheral emboli and help distinguish true emboli from artifacts; (iii) Reduced-dose protocols: Development of CTPA protocols that maintain diagnostic accuracy while reducing radiation exposure would facilitate serial imaging for surveillance when indicated; (iv) Alternative imaging modalities: Novel approaches such as magnetic resonance pulmonary angiography or ventilation-perfusion single-photon emission CT may offer alternatives to CTPA with different diagnostic performance characteristics.

## Conclusions

The widespread adoption of multidetector CTPA has transformed PE diagnosis by enabling detection of subsegmental emboli that would have been missed by earlier imaging technologies, creating a clinical paradox of rising diagnosis rates alongside declining case-fatality rates. Current evidence demonstrates low short-term recurrence rates after isolated SSPE that do not differ significantly from more proximal PE, yet anticoagulation carries nontrivial bleeding risks, establishing clinical equipoise about net benefit in low-risk cases. Diagnostic challenges, including substantial inter-observer variability and the potential for false-positive interpretations, compound management uncertainty.

For internists, a risk-stratified approach is recommended: higher-risk patients should generally receive anticoagulation, while carefully selected low-risk patients may be reasonable candidates for clinical observation with close follow-up. Shared decision-making that explicitly discusses the uncertain evidence base, recurrence risk, bleeding risk, and individual patient values is essential. Ongoing randomized trials will provide the first high-quality evidence to guide anticoagulation decisions for isolated SSPE. Until these results are available, management must balance the potential harms of overtreatment against the potential harms of undertreatment, with rigorous research and patient-centered care essential to optimize outcomes.

## References

[1] Triggiani S, Pellegrino G, Mortellaro S (2025). Comprehensive review of pulmonary embolism imaging: past, present and future innovations in computed tomography (CT) and other diagnostic techniques. Jpn J Radiol.

[2] Abdi S, Naderi N, Zakariaee SS (2025). Diagnostic performance of quantitative computed tomography pulmonary angiography parameters in patients with pulmonary embolism. Pulm Med.

[3] Bushra F, Chowdhury ME, Sarmun R (2024). Deep learning in computed tomography pulmonary angiography imaging: a dual-pronged approach for pulmonary embolism detection. Expert Syst Appl.

[4] den Exter PL, Kroft LJ, Gonsalves C (2020). Establishing diagnostic criteria and treatment of subsegmental pulmonary embolism: a Delphi analysis of experts. Res Pract Thromb Haemost.

[5] Peiman S, Abbasi M, Allameh SF, Asadi Gharabaghi M, Abtahi H, Safavi E (2016). Subsegmental pulmonary embolism: a narrative review. Thromb Res.

[6] de Jong CM, Kroft LJ, van Mens TE, Huisman MV, Stöger JL, Klok FA (2024). Modern imaging of acute pulmonary embolism. Thromb Res.

[7] Lasserson D, Gaddu P, Mehta S (2024). Stopping anticoagulation for isolated or incidental pulmonary embolism: the STOPAPE RCT protocol. Health Technol Assess.

[8] Le Gal G, Kovacs MJ, Bertoletti L (2022). Risk for recurrent venous thromboembolism in patients with subsegmental pulmonary embolism managed without anticoagulation: a multicenter prospective cohort study. Ann Intern Med.

[9] Girardi L, Ciuffini LA, Mai V (2024). Risk of recurrent venous thromboembolism and bleeding in patients with acute isolated subsegmental pulmonary embolism. Thromb Res.

[10] Hirao-Try Y, Vlazny DT, Meverden R (2022). Single versus multiple and incidental versus symptomatic subsegmental pulmonary embolism: clinical characteristics and outcome. J Thromb Thrombolysis.

[11] Brin D, Gilat EK, Raskin D, Goitein O (2025). Evaluation of AI-based detection of incidental pulmonary emboli in cardiac CT angiography scans. Int J Cardiovasc Imaging.

[12] Wada N, Li Y, Gagne S (2023). Incidence and severity of pulmonary embolism in COVID-19 infection: Ancestral, Alpha, Delta, and Omicron variants. Medicine (Baltimore).

[13] Qdaisat A, Wu CC, Yeung SJ (2019). Normal D-dimer levels in cancer patients with radiologic evidence of pulmonary embolism. J Thromb Thrombolysis.

[14] Bariteau A, Stewart LK, Emmett TW, Kline JA (2018). Systematic review and meta‐analysis of outcomes of patients with subsegmental pulmonary embolism with and without anticoagulation treatment. Acad Emerg Med.

[15] Ghani H, Weir-McCall JR, Ruggiero A, Pepke-Zaba J (2024). Imaging in chronic thromboembolic pulmonary disease: current practice and advances. Int J Cardiol Congenit Heart Dis.

[16] Lysdahlgaard S, Hess S, Gerke O, Weber Kusk M (2020). A systematic literature review and meta-analysis of spectral CT compared to scintigraphy in the diagnosis of acute and chronic pulmonary embolisms. Eur Radiol.

[17] Cellina M, Pavan M, Finardi N (2025). Advancements in acute pulmonary embolism diagnosis and treatment: a narrative review of emerging imaging techniques and intravascular interventions. J Cardiovasc Dev Dis.

[18] Kligerman SJ, Mitchell JW, Sechrist JW, Meeks AK, Galvin JR, White CS (2018). Radiologist performance in the detection of pulmonary embolism: features that favor correct interpretation and risk factors for errors. J Thorac Imaging.

[19] Swan D, Hitchen S, Klok FA, Thachil J (2019). The problem of under-diagnosis and over-diagnosis of pulmonary embolism. Thromb Res.

[20] Newnham M, Turner AM (2019). Diagnosis and treatment of subsegmental pulmonary embolism. World J Respirol.

[21] Hutchinson BD, Navin P, Marom EM, Truong MT, Bruzzi JF (2015). Overdiagnosis of pulmonary embolism by pulmonary CT angiography. AJR Am J Roentgenol.

[22] Sharma S, Lucas CD (2017). Increasing use of CTPA for the investigation of suspected pulmonary embolism. Postgrad Med.

[23] Sohns C, Amarteifio E, Sossalla S, Heuser M, Obenauer S (2008). 64-Multidetector-row spiral CT in pulmonary embolism with emphasis on incidental findings. Clin Imaging.

[24] Hinrichs JB, von Falck C, Hoeper MM, Olsson KM, Wacker FK, Meyer BC, Renne J (2016). Pulmonary artery imaging in patients with chronic thromboembolic pulmonary hypertension: comparison of cone-beam CT and 64-row multidetector CT. J Vasc Interv Radiol.

[25] Armitage MN, Mughal AZ, Huntley CC, Lasserson D, Newnham M (2023). A multicentre observational study of the prevalence, management, and outcomes of subsegmental pulmonary embolism. J Thromb Thrombolysis.

[26] Long B, Koyfman A (2017). Best clinical practice: current controversies in pulmonary embolism imaging and treatment of subsegmental thromboembolic disease. J Emerg Med.

[27] Baumgartner C, Tritschler T (2021). Clinical significance of subsegmental pulmonary embolism: an ongoing controversy. Res Pract Thromb Haemost.

[28] Baumgartner C, Klok FA, Carrier M (2020). Clinical Surveillance vs. Anticoagulation For low-risk patiEnts with isolated SubSegmental Pulmonary Embolism: protocol for a multicentre randomised placebo-controlled non-inferiority trial (SAFE-SSPE). BMJ Open.

[29] Ayobi A, Chang PD, Chow DS (2024). Performance and clinical utility of an artificial intelligence-enabled tool for pulmonary embolism detection. Clin Imaging.

[30] Li L, Peng M, Zou Y, Li Y, Qiao P (2025). The promise and limitations of artificial intelligence in CTPA-based pulmonary embolism detection. Front Med (Lausanne).

[31] Farzaei A, Hajzeinolabedini F, Sharif Kashani B (2025). The role of artificial intelligence in diagnosing pulmonary embolism: a systematic review and meta-analysis. Arch Acad Emerg Med.

[32] Nicoletto M, Maino A, Guarascio M, Cavalleri MA, Gallucci F, Dentali F, Pomero F (2026). Anticoagulation therapy vs clinical surveillance in isolated subsegmental pulmonary embolism: a systematic review and meta-analysis. Thromb Res.

[33] Banerjee I, Chen MC, Lungren MP, Rubin DL (2018). Radiology report annotation using intelligent word embeddings: applied to multi-institutional chest CT cohort. J Biomed Inform.

[34] Goy J, Lee J, Levine O, Chaudhry S, Crowther M (2015). Sub-segmental pulmonary embolism in three academic teaching hospitals: a review of management and outcomes. J Thromb Haemost.

[35] Li J, Rolfe-Vyson V, Rowland V, Woulfe T, Merriman E (2023). Management of single subsegmental pulmonary embolism: a prospective observational study at North Shore and Waitakere Hospitals, Auckland. Intern Med J.

[36] Stoller N, Limacher A, Méan M (2019). Clinical presentation and outcomes in elderly patients with symptomatic isolated subsegmental pulmonary embolism. Thromb Res.

[37] Sharma Y, Sumanadasa S, Shahi R, Horwood C, Thompson C (2023). The value of distinguishing patients with isolated subsegmental pulmonary embolism presenting to two tertiary hospitals in Australia: an observational study. J Thromb Thrombolysis.

[38] Pallazola VA, Kapoor RK, Kapoor K, McEvoy JW, Blumenthal RS, Gluckman TJ (2019). Anticoagulation risk assessment for patients with non-valvular atrial fibrillation and venous thromboembolism: a clinical review. Vasc Med.

[39] Barker RC, Marval P (2011). Venous thromboembolism: risks and prevention. Contin Educ Anaesth Crit Care Pain.

[40] Blann AD, Lip GY (2016). Non-vitamin K antagonist oral anticoagulants (NOACs) for the management of venous thromboembolism. Heart.

[41] Yoo HH, Queluz TH, El Dib R (2014). Anticoagulant treatment for subsegmental pulmonary embolism. Cochrane Database Syst Rev.

[42] Konstantinides S, Goldhaber SZ (2012). Pulmonary embolism: risk assessment and management. Eur Heart J.

[43] Zuin M (2025). How important is measuring hemodynamic parameters for risk stratification in acute pulmonary embolism patients?. Expert Rev Cardiovasc Ther.

[44] Zuin M, Henkin S, Harder EM, Piazza G (2024). Optimal hemodynamic parameters for risk stratification in acute pulmonary embolism patients. J Thromb Thrombolysis.

[45] Latsios G, Mantzouranis E, Kachrimanidis I (2025). Recent advances in risk stratification and treatment of acute pulmonary embolism. World J Cardiol.

[46] Peracaula M, Sebastian L, Francisco I, Vilaplana MB, Rodríguez-Chiaradía DA, Tura-Ceide O (2024). Decoding pulmonary embolism: pathophysiology, diagnosis, and treatment. Biomedicines.

[47] Khorana AA, Mackman N, Falanga A (2022). Cancer-associated venous thromboembolism. Nat Rev Dis Primers.

[48] Sharma K (2025). Management of cancer-associated thrombosis and related complications. Deep Learning and Computer Vision: Models and Biomedical Applications: Volume 1.

[49] Bianchi L, Ghigliotti G, Sarocchi M, Canale C, Toma M, Porto I, Spallarossa P (2026). Practical considerations about management of pulmonary embolism in patients with cancer. J Thromb Thrombolysis.

[50] Makowska A, Treumann T, Venturini S, Christ M (2024). Pulmonary embolism in pregnancy: a review for clinical practitioners. J Clin Med.

[51] Nauffal D, Ballester M, Reyes RL, Jiménez D, Otero R, Quintavalla R, Monreal M (2012). Influence of recent immobilization and recent surgery on mortality in patients with pulmonary embolism. J Thromb Haemost.

[52] Angriman F, Ferreyro BL, Posadas-Martinez ML, Giunta D, Vazquez FJ, Vollmer WM (2015). Wells score and poor outcomes among adult patients with subsegmental pulmonary embolism: a cohort study. Clin Appl Thromb Hemost.

[53] Inácio Cazeiro D, Azaredo Raposo M, Guimarães T (2025). Chronic thromboembolic pulmonary hypertension: a comprehensive review of pathogenesis, diagnosis, and treatment strategies. Rev Port Cardiol.

[54] Luijten D, de Jong CM, Ninaber MK, Spruit MA, Huisman MV, Klok FA (2023). Post-pulmonary embolism syndrome and functional outcomes after acute pulmonary embolism. Semin Thromb Hemost.

[55] Morris TA, Fernandes TM, Channick RN (2023). Evaluation of dyspnea and exercise intolerance after acute pulmonary embolism. Chest.

[56] Chen M, Wang Y, Zhou T (2024). Effect of exercise on physical fitness and quality of life in patients with pulmonary embolism: a systematic review and meta-analysis. J Thorac Dis.

[57] Yang D, Deng J, Huang S, He H, Dai C, Zhao D, Chen C (2026). Risk factors and outcomes of subsegmental versus more central pulmonary embolism in patients with lung cancer. Thromb Haemost.

